# Conjugation of Lectin to Poly(ε-caprolactone)-*block*-glycopolymer Micelles for In Vitro Intravesical Drug Delivery

**DOI:** 10.3390/polym8110379

**Published:** 2016-10-26

**Authors:** Ning Ning Li, Xiao Yan Cai, Jiu Cun Chen, Xue Feng Hu, Li Qun Xu

**Affiliations:** 1 Institute for Clean Energy and Advanced Materials, Faculty of Materials and Energy, Southwest University, Chongqing 400715, China; lny920047@163.com (N.N.L.); cxy921211@163.com (X.Y.C.); chenjc@swu.edu.cn (J.C.C.); 2 National Engineering Research Center for Biomaterials, Sichuan University, Chengdu 610064, China

**Keywords:** lectin, glycopolymer, mucoadhesion, reactive pentafluorophenyl ester, bladder cancer, micelles

## Abstract

Amphiphilic poly(ε-caprolactone)-*block*-poly[2-(α-d-mannopyranosyloxy) ethyl acrylamide] (PCL-*b*-PManEA) block copolymers were synthesized via a combination of ring-opening polymerization (ROP), reversible addition-fragmentation chain transfer (RAFT) polymerization and reactive ester-amine reaction. The PCL-*b*-PManEA block copolymers can self-assemble into micelles and encapsulate anticancer drug doxorubicin (DOX). To enhance mucoadhesive property of the resulting DOX-loaded PCL-*b*-PManEA micelles, Concanavalin A (ConA) lectin was further conjugated with the micelles. Turbidimetric assay using mucin shows that the DOX-loaded PCL-*b*-PManEA@ConA micelles are mucoadhesive. DOX release from the DOX-loaded PCL-*b*-PManEA@ConA micelles in artificial urine at 37 °C exhibits an initial burst release, followed by a sustained and slow release over three days. Confocal laser scanning microscope (CLSM) images indicate that the DOX-loaded PCL-*b*-PManEA@ConA micelles can be effectively internalized by UMUC3 human urothelial carcinoma cells. The DOX-loaded PCL-*b*-PManEA@ConA micelles exhibit significant cytotoxicity to these cells.

## 1. Introduction

Bladder cancer is one of the most common malignancies in the world [1,2]. Intravesical chemotherapy via infusion of drugs through urethra into bladder is the most common method for treating early state bladder cancer [1]. However, most of the intravesical drugs are typically maintained intravesically for 2 h, due to voidage of urine after instillation [3]. The lack of success of intravesical chemotherapy is probably due in part to the short drug dwelling time in the bladder and the low drug permeability of the bladder wall [4].

The presence of the mucosal layer on the urothelial surface could be utilized to increase the intravesical drug dwelling time by exploiting the mucoadhesion phenomenon. Mucoadhesion, defined as the ability to adhere to the mucosa-gel layer, is a key element in the development of effective drug delivery systems [5]. The mucoadhesive ability of a dosage form depends on the natural properties of mucosal tissue and the physicochemical properties of the polymeric formulation [6]. Several classes of polymeric materials have been proposed to be mucoadhesive due to their ability to conjugate with the mucosae via non-covalent interactions, such as hydrogen bonds, van-der Waals forces and ionic interactions [7,8]. Much effort has been made to enhance the mucoadhesive properties of polymeric formulation via formation of covalent bonds, such as disulfide linkage between the polymer and mucosa [9,10]. Mucoadhesive techniques have further evolved into more specific systems based on lectins [11]. Lectins are proteins or glycoproteins of non-immunological origin, which can bind to specific carbohydrate residues, such as glucose/mannose, *N*-acetyl-glucosamine, *N*-acetyl-galactosamine/galactose, l-fucose and sialic acid [12]. These carbohydrate groups are present in glycoproteins of mammalian mucosal layers [13]. Thus, lectin-functionalized formulations could provide specific biological interaction to promote mucoadhesion to the mucosal layer [14,15].

**Scheme 1:**
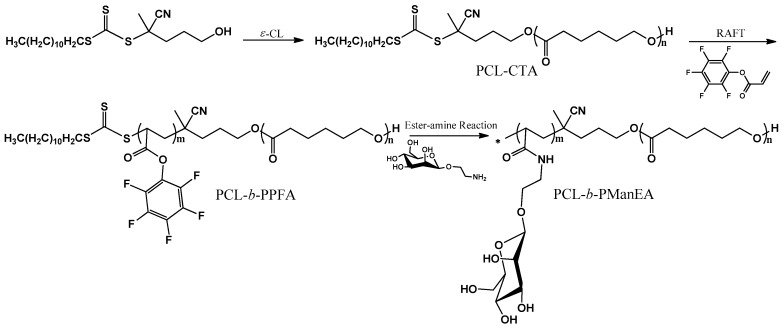
Synthetic route of poly(ε-caprolactone)-*block*-poly[2-(α-d-mannopyranosyloxy) ethyl acrylamide] (PCL-*b*-PManEA) block copolymer.

**Scheme 2:**
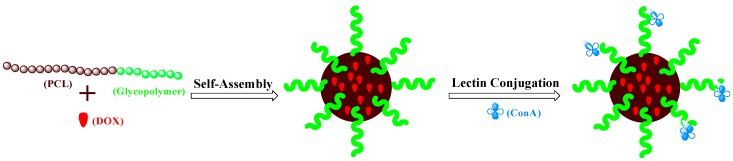
Self-assembly of PCL-*b*-PManEA block copolymer into micellar nanoparticle and its conjugation with Concanavalin A (ConA) lectin.

The goal of this work was to synthesize lectin-functionlizable drug-loaded micelles for the intravesical drug delivery. Glycopolymer-*block*-poly(ε-caprolactone) (PCL) copolymers are good choices for this system, as the PCL block can self-assemble in aqueous media and encapsulate anti-cancer drugs and the glycopolymer can conjugate with lectins. Glycopolymer-*block*-PCL copolymer has been prepared via post-modification of the poly(acryloyl cyclic carbonate)-*block*-PCL copolymer with thiolated lactobionic acid [16]. It has also been synthesized via atom transfer radical polymerization of unprotected glycomonomer using four-arm PCL as the macroinitiator [17]. Herein, amphiphilic PCL-*block*-poly[2-(α-d-mannopyranosyloxy) ethyl acrylamide] (PCL-*b*-PManEA) block copolymers were prepared via the combination of ring-opening polymerization (ROP), reversible addition-fragmentation chain transfer (RAFT) polymerization and reactive ester-amine reaction. The biodegradable and hydrophobic PCL block was synthesized via ROP of ε-caprolactone. The biocompatible and hydrophilic glycopolymer block was prepared by nucleophilic substitution of the reactive pentafluorophenyl ester group with 2-aminoethyl-α-d-mannopyranoside (Scheme 1). The doxorubicin (DOX)-loaded micelles were formed by self-assembly of amphiphilic PCL-*b*-PManEA block copolymers in aqueous solution and encapsulation of DOX in the micelles. The resulting DOX-loaded PCL-*b*-PManEA micelles can specifically bind to Concanavalin A (ConA) via lectin-carbohydrate interaction (Scheme 2). After conjugation with the DOX-loaded PCL-*b*-PManEA micelles, the tetravalent ConA still possesses active sites for further complexation with carbohydrate residues on the mucosal layer. The mucoadhesive properties of the as-synthesized DOX-loaded PCL-*b*-PManEA@ConA micelles can thus be enhanced. The in vitro drug release, mucoadhesive property, cytotoxicity and cellular uptake by UMUC3 human urothelial carcinoma cells of these DOX-loaded PCL-*b*-PManEA@ConA micelles were investigated.

## 2. Materials and Methods

### 2.1. Materials

4-Cyano-4-[(dodecylsulfanylthiocarbonyl)sulfanyl]pentanol (CDSP, containing 15 wt % ethyl acetate as residual solvent), tin(II) 2-ethylhexanoate (Sn(Oct)_2_, 95%), ε-caprolactone (ε-CL, 99%), concanavalin A (ConA, Type VI, lyophilized powder) lectin, mucin from porcine stomach (type II) and 2,2′-azoisobutyronitrile (AIBN, 98%) were purchased from Sigma-Aldrich Chemical Co (St. Louis, MO, USA), Doxorubicin (DOX) hydrochloric acid salt (DOX·HCl, 99%) was purchased from LC Laboratories (Woburn, MA, USA). All other regents were purchased from Sigma-Aldrich or Merck Chem. Co. (Darmstadt, Germany), and were used without further purification. Pentafluorophenyl acrylate (PFA) and 2-aminoethyl-α-d-mannopyranoside were synthesized according to the methods reported in the literature [18,19].

### 2.2. Synthesis of Poly(ε-caprolactone) (PCL) Macro-Chain Transfer Agent (Macro-CTA)

PCL macro-CTA was synthesized by ring-opening polymerization (ROP) of ε-CL using Sn(Oct)_2_ as a catalyst and CDSP as an initiator. CDSP (200.0 mg, 5.1 × 10^−4^ mol) and ε-CL (5.6 mL, 5.1 × 10^−2^ mol) were charged into a 50-mL Schlenk flask equipped with a magnetic stirring bar. A solution of Sn(Oct)_2_ (40.5 mg, 1.0 × 10^−4^ mol) in 2 mL of toluene was also added using a syringe. The reaction mixture was degassed with argon for 20 min, sealed, and stirred at 110 °C for 12 h. The flask was then immersed in an ice bath to stop the reaction. The resulting solid was dissolved with 10 mL of tetrahydrofuran (THF) and then poured into 200 mL of cold diethyl ether to precipitate the polymer. The precipitate was filtered, followed by drying under reduced pressure at room temperature for 24 h. The resulting PCL samples prepared from (CDSP):(ε-CL) molar feed ratios of 1:100 and 1:150 are referred to as PCL1 and PCL2, respectively. The number-average molecular weights (*M*_n_) of PCL1 and PCL2 were determined from gel permeation chromatography (GPC, Waters Co., Milford, MA, USA) analysis to be 8800 and 12,800 g/mol, respectively.

### 2.3. Reversible Addition-Fragmentation Chain Transfer (RAFT) Polymerization of PFA Using PCL as the Macro-CTA

The PCL macro-CTA (PCL1, 1.3 g, 1.5 × 10^−4^ mol; or PCL2, 1.9 g, 1.5 × 10^−4^ mol) was first dissolved in 8 mL of toluene in a 25 mL round-bottom flask under magnetic stirring. Subsequently, AIBN (6.2 mg, 3.8 × 10^−5^ mol) and PFA (1.8 g, 7.5 × 10^−3^ mol) were added into the above mixture under vigorous stirring. The reaction mixture was degassed with argon for 30 min. The flask was sealed and kept in a 70 °C oil bath under stirring for 12 h. The reaction mixture was then cooled in an ice bath, diluted with 10 mL of THF, and precipitated into 200 mL of cold ethanol. The precipitate was filtered, washed with ethanol, followed by drying under reduced pressure at room temperature for 24 h. The obtained PCL-*block*-poly(PFA) (PCL-*b*-PPFA) block copolymers from PCL1 macro-CTA and PCL2 macro-CTA are referred to as PCL1-*b*-PPFA and PCL2-*b*-PPFA, respectively. The *M*_n_s of PCL1-*b*-PPFA and PCL2-*b*-PPFA were determined from GPC analysis to be 12,500 and 16,100 g/mol, respectively.

### 2.4. Synthesis of PCL-block-poly[2-(α-d-mannopyranosyloxy) ethyl acrylamide] (PCL-b-PManEA) Block Copolymers

PCL-*b*-PManEA block copolymers were prepared by post-functionalization of the respective PCL-*b*-PPFA block copolymers with 2-aminoethyl-α-d-mannopyranoside via reactive ester-amine reaction. The PCL1-*b*-PPFA block copolymer (1.0 g, 1.3 × 10^−3^ mol of PFA repeat units), triethylamine (0.3 mL, 2.1 × 10^−3^ mol), 2-aminoethyl-α-d-mannopyranoside (150 mol % relative to PFA repeat units, 0.6 g, 2.1 × 10^−3^ mol) and dry *N*,*N*-dimethylformamide (DMF, 10 mL) were added into a 25 mL round-bottom flask. Excess 2-aminoethyl-α-d-mannopyranoside was used to ensure the complete conversion of pentafluorophenyl ester groups. The reaction mixture was stirred at 50 °C for 24 h under an argon atmosphere. After that, the flask was cooled down to room temperature and the polymer was precipitated into 200 mL of cold ethanol. The obtained polymer was filtered and re-dissolved in 20 mL of DMF. The re-dissolution and precipitation process was repeated three times to remove any residual reactant adducts. About 0.7 g of the PCL1-*b*-PManEA copolymer was obtained after drying under reduced pressure at room temperature for 24 h. The corresponding sample prepared from PCL2-*b*-PPFA block copolymer is referred to as PCL2-*b*-PManEA block copolymer.

### 2.5. Preparation of DOX-Loaded PCL-b-PManEA Micelles

DOX·HCl (10 mg), triethylamine (5.0 µL) and PCL-*b*-PManEA block copolymer (100 mg) were dissolved in 10 mL of DMF. The mixture was stirred at 25 °C for 2 h, and then added dropwise to 50 mL of doubly distilled water under vigorous stirring at 25 °C. The solution was further sonicated for 10 min and stirred at 25 °C for 4 h. After that, the micelle solution was dialyzed against doubly distilled water (molecular weight cutoff = 3500 Da) for 24 h. During dialysis, the water was renewed every 4 h. The resulting DOX-loaded PCL-*b*-PManEA micelles from PCL1-*b*-PManEA and PCL2-*b*-PManEA block copolymers are referred to as DOX-loaded PCL1-*b*-PManEA and PCL2-*b*-PManEA micelles, respectively. For determination of drug loading, the DOX-loaded PCL-*b*-PManEA micelles were analyzed by UV-visible absorption spectroscopy (Hitachi U2800 spectrophotometer, Hitachi Ltd., Tokyo, Japan) at the wavelength of 480 nm. The calibration curve (*y* (absorption intensity) = 17.341 × *x* (mg/mL) + 0.004) was obtained using DOX·HCl aqueous solutions of different concentrations. The drug loadings of DOX-loaded PCL1-*b*-PManEA and PCL2-*b*-PManEA micelles were determined to be 3.60 and 3.61 wt %, respectively. Solid DOX-loaded PCL-*b*-PManEA micelles were obtained by freeze-drying. DOX-free PCL1-*b*-PManEA micelles were prepared via a similar procedure in the absence of DOX and triethylamine.

### 2.6. Preparation of DOX-Loaded PCL-b-PManEA Micelles-ConA Conjugates (DOX-Loaded PCL-b-PManEA@ConA Micelles)

DOX-loaded PCL-*b*-PManEA micelles (30 mg) were dispersed in 30 mL of doubly distilled water in a 50 mL round-bottom flask with the aid of sonication for 2 min. An aqueous solution of 1.5 mg/mL of ConA was prepared, and 4 mL of the ConA solution was transferred into the flask. The solution in the flask was thoroughly mixed via vigorous stirring for 15 min and immediately returned to the freeze-dryer to obtain the solid DOX-loaded PCL-*b*-PManEA@ConA micelles. The respective DOX-loaded PCL1-*b*-PManEA and PCL2-*b*-PManEA micelles-ConA conjugates are referred to as DOX-loaded PCL1-*b*-PManEA@ConA and PCL2-*b*-PManEA@ConA micelles. DOX-free PCL1-*b*-PManEA@ConA micelles were prepared from DOX-free PCL1-*b*-PManEA micelles in a similar manner.

### 2.7. In Vitro Drug Release

Fifteen milligrams of DOX-loaded PCL-*b*-PManEA@ConA micelles were dispersed in 15 mL of respective phosphate buffered saline (PBS, pH = 7.4) and artificial urine (AU, pH = 6.1). AU was prepared according to the method reported in the literature [20]. Ten milliliters of the respective DOX-loaded PCL-*b*-PManEA@ConA micelles dispersions were transferred into dialysis bags with a molecular weight cut-off of 3500 Daltons (Spectrum Labs). The dialysis bags were then immersed into 10 mL of the corresponding media (PBS or AU) in 50-mL centrifuge tubes with gentle shaking (100 rpm) at 37 °C. At predetermined time intervals, 10 mL of the media outside the dialysis bags were extracted, and were replaced by fresh media. The release experiments were conducted for 72 h in PBS and AU. The released DOX amounts were determined by UV-visible absorption spectroscopy based on the absorption intensity at the wavelength of 480 nm for samples in PBS and AU. The calibration curves for DOX in PBS and AU were (*y* (absorption intensity) = 14.794 × *x* (mg/mL) − 0.002) and (*y* (absorption intensity) = 10.194 × *x* (mg/mL) + 0.003), respectively.

### 2.8. Cell Culture and Incubation

The UMUC3 human urothelial carcinoma cell line, purchased from American Type Culture Collection (ATCC, Manassas, VA, USA), were cultivated in Dulbecco’s modified Eagle’s medium (DMEM) at 37 °C in a humidified environment of 5% CO_2_. The medium was changed every two days until cell confluence was reached.

### 2.9. Cell Viability

The cytotoxicities of free DOX, DOX-free and DOX-loaded PCL1-*b*-PManEA@ConA micelles towards UMUC3 cells were determined from the assay based on the reduction of 3-(4,5-dimethylthiazol-2-yl)-2,5-diphenyltetrazolium bromide (MTT) reagent. The MTT assay was performed in a 96-well plate following the standard procedure with minor modifications. The cells were seeded at a density of 3000 cells per well and incubated at 37 °C for 24 h. The medium was then replaced by one containing free DOX with drug concentrations ranging from 1.5 × 10^−3^ to 100 μg/mL or DOX-loaded PCL-*b*-PManEA@ConA micelles with micelles concentrations ranging from 3.0 × 10^−5^ to 2.0 mg/mL for 2 h. Control experiments were carried out using the complete growth culture medium, without free DOX or micelles. After that, the culture medium in each well was removed. The cells were washed thrice with PBS, and 100 μL of fresh culture medium was added into each well. After 72 h of incubation at 37 °C, the medium was removed, and 100 µL of MTT solution (0.5 mg/mL in DMEM medium) were added to each well. After an additional 4 h of incubation, culture supernatants were aspirated, and the formazan crystals were dissolved with 100 µL of dimethyl sulfoxide (DMSO) for 15 min. The optical absorbance was then measured at 600 nm on a microplate reader (Tecan GENios, Tecan Asia Ltd., Grödig, Austria). The results were expressed as percentages relative to that obtained in the control experiment.

### 2.10. Cellular Uptake

For cellular uptake experiments, UMUC3 cells were seeded on glass dishes at a density of 5 × 10^4^ cells for 24 h. The medium was then aspirated and replaced with media containing DOX-loaded PCL1-*b*-PManEA@ConA micelles at a concentration of 2 mg/mL in the absence and presence of 1-methyl α-d-mannopyranoside (MeMan, 20 mM). The cells were cultured for another 2 h, and washed thrice with sterilized doubly distilled water to remove the physically adsorbed DOX-loaded PCL1-*b*-PManEA@ConA micelles completely. After that, the cells were fixed in formaldehyde aqueous solution (4%, *v*/*v*) for 30 min. Following fixation, the cells were observed on a Nikon (A1) Confocal Laser Scanning Microscope (CLSM, Nikon, Tokyo, Japan). For fluorescence imaging, the cells were excited at 488 nm, and the fluorescence in the wavelength region of 570–620 nm was recorded.

### 2.11. Mucoadhesive Property of DOX-loaded PCL1-b-PManEA@ConA Micelles

The mucoadhesive property of DOX-loaded PCL1-*b*-PManEA@ConA micelles was evaluated using the turbidimetric assay. One milligram per milliliter of mucin from porcine stomach was first prepared in phosphate-buffered saline (PBS, pH = 7.4). One and half milliliters of this mucin solution was transferred into a quartz cell and placed into the holding block of the spectrometer (Hitachi U2800 spectrophotometer). One and half milliliters of the DOX-loaded PCL1-*b*-PManEA micelles, or DOX-loaded PCL1-*b*-PManEA@ConA micelles (1 mg/mL in PBS, pH = 7.4), was added into the quartz cell containing the mucin solution. The mixture in the quartz cell was mixed using a vortex mixer and immediately returned to the holding block, where the transmittance at 420 nm was continuously monitored for 2 h. The starting transmittance of each sample was set as 100%. The controlled experiment was carried out using mucin, DOX-loaded PCL1-*b*-PManEA micelles or DOX-loaded PCL1-*b*-PManEA@ConA micelles (0.5 mg/mL) only. In the absence of mucin, the turbidity of DOX-loaded PCL1-*b*-PManEA micelles and DOX-loaded PCL1-*b*-PManEA@ConA micelles changed by less than 2% after 1 day (data not shown). The competitive binding assays for free ConA (0.5 mg/mL) and DOX-loaded PCL1-*b*-PManEA@ConA micelles (1 mg/mL) were carried out in the presence of MeMan (20 mM).

### 2.12. Characterization

The chemical structures of obtained polymers were characterized by ^1^H NMR spectroscopy on a Bruker DRX 400 MHz spectrometer (Bruker UK Ltd., Coventry, UK). Gel permeation chromatography (GPC) was performed on a Waters GPC system, equipped with a Waters 1515 isocratic HPLC pump, a Waters 717 plus Autosampler injector, a Waters 2414 refractive index detector and a PLgel 10 µm Mixed-B column (Agilent Technologies, S/N 10M-MB-D7-9K8), using THF as the eluent at a flow rate of 1.0 mL/min at 35 °C. X-ray photoelectron spectroscopy (XPS) measurements were carried out on a Kratos AXIS Ultra HSA spectrometer (Kratos Analytical Ltd., Manchester, UK) equipped with a monochromatized AlKα X-ray source (1468.71 eV photons). Field-emission transmission electron microscopy (FETEM) images were obtained from a JEOL JEM-2010 FETEM (JEOL Ltd., Tokyo, Japan). Aqueous samples were drop-casted on copper TEM grids, followed by drying under reduced pressure for 12 h. FT-IR spectroscopy analysis was carried out on a Bio-Rad FTS-135 spectrophotometer (Bio-Rad Laboratories Inc., Cambridge, MA, USA). Dynamic light scattering (DLS) measurements were performed on a Brookhaven 90 plus laser light scattering spectrometer (Brookhaven Instruments Co., Holtsville, NY, USA) at a scattering angle of 90°. Aqueous samples were sonicated in the water bath for 2 min. Loaded samples were then diluted by doubly distilled water to make sure that the particles count rate was within the range of 200–700 kcps. For every sample, five runs were conducted. The measurement was carried out at 25 °C with the final results being given as the average of the five analyses for each sample plus its standard derivation. The effective diameters were calculated by ZetaPlus Particle Sizing Software Version 3.93 (Brookhaven Instruments Co., Holtsville, NY, USA).

## 3. Results and Discussion

### 3.1. Synthesis of PCL-b-PManEA Block Copolymers

There are many methods for the synthesis of homo/block glycopolymers, including conventional radical polymerization, living ionic polymerization, ring-opening metathesis polymerization (ROMP) and controlled radical polymerization (CRP) [21,22]. These techniques usually include procedures for the protection of glycomonomers and deprotection of the resulting homo/block glycopolymers [23]. To simplify the synthetic approach to glycopolymers, post-functionalization of a preformed polymer/copolymer using active sugar moieties is desirable. The active pentafluorophenyl esters are advantageous for post-modification [24,25,26], and will be suitable for building up the glycopolymer structures. In the present work, the combination of ROP, RAFT polymerization and reactive ester-amine reaction was utilized to synthesize the PCL-*b*-PManEA block copolymers. The detailed synthesis route, which consists of three consecutive steps, was shown in Scheme 1.

**Figure 1:**
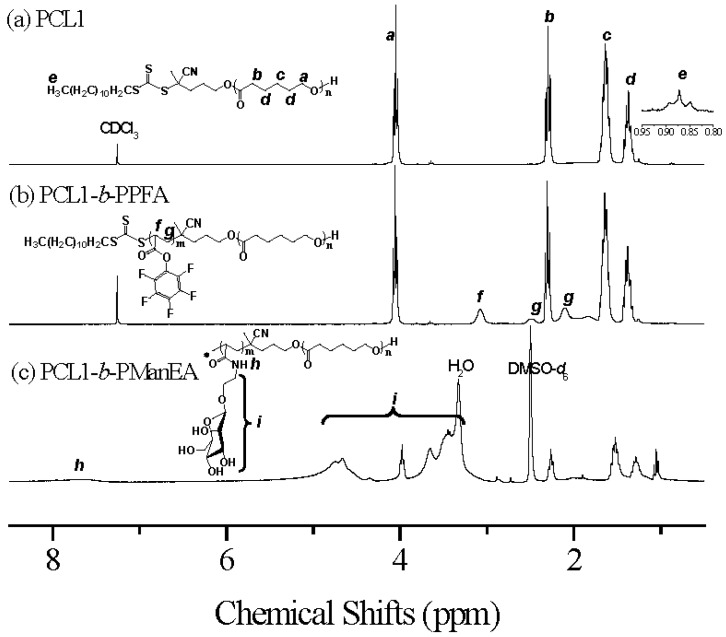
^1^H NMR spectra of: (**a**) PCL1 homopolymer; (**b**) PCL1-*b*-PPFA; and (**c**) PCL1-*b*-PManEA block copolymers.

PCL macro-CTA was synthesized via ROP of ε-CL using CDSP as the initiator and Sn(Oct)_2_ as the catalyst. The resulting PCL macro-CTAs prepared from (CDSP):(ε-CL) molar feed ratios of 1:100 and 1:150 are referred to as PCL1 and PCL2, respectively. The *M*_n_s of PCL1 and PCL2, as determined by GPC, were 8800 and 12,800 g/mol respectively, with a polydispersity index (PDI) of about 1.50 (Supplementary Materials Table S1 and Figure S1). In the future work, other catalysts should be chosen to obtain the PCL homopolymers with narrow molecular weight distributions and good chain extension efficiency. Figure 1a shows the ^1^H NMR spectrum of PCL1 in CDCl_3_. The chemical shifts at 4.05, 2.30, 1.64 and 1.37 ppm are characteristic signals of PCL repeating units [27]. In addition, the chemical shift at about 0.90 ppm is assigned to the methyl proton of CDSP, indicating that the CTA’s persist in the PCL samples.

**Figure 2:**
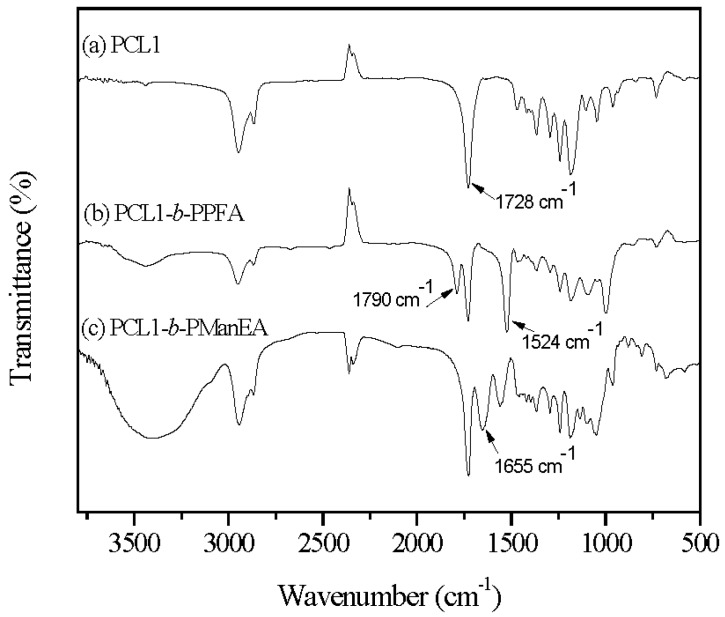
FT-IR spectra of: (**a**) PCL1 homopolymer; (**b**) PCL1-*b*-PPFA; and (**c**) PCL1-*b*-PManEA block copolymers.

**Figure 3:**
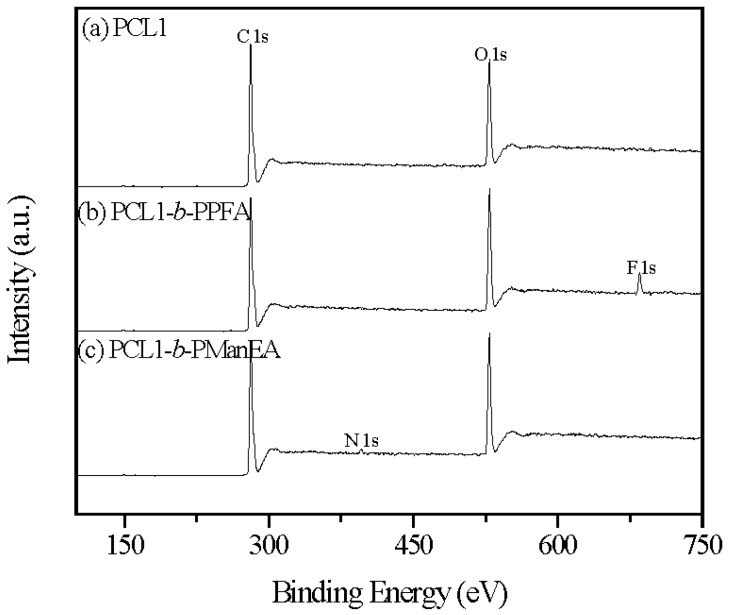
X-ray photoelectron spectroscopy (XPS) wide-scan spectra of: (**a**) PCL1 homopolymer; (**b**) PCL1-*b*-PPFA; and (**c**) PCL1-*b*-PManEA block copolymers.

These PCL samples were then utilized as macro-CTAs to mediate RAFT polymerization of PFA, using AIBN as the initiator. Successful block copolymerization of PFA was confirmed by GPC, ^1^H NMR spectroscopy, FT-IR spectroscopy and XPS measurement. The GPC elution curves of PCL1-*b*-PPFA and PCL2-*b*-PPFA are shown in Figure S1 (Supplementary Materials). The respective *M*_n_ has increased substantially after block copolymerization, as indicated by the shifting of the GPC elution curve to shorter time. The ^1^H NMR spectrum of PCL1-*b*-PPFA (Figure 1b) shows the resonances of methine and methylene protons on the backbone of PPFA. These results indicate that the PFA monomers have been successfully copolymerized. From the integrated area ratio of the signal due to the methylene group in PCL repeating units at 4.05 ppm and the signal due to the methine group in PPFA backbone at 3.07 ppm, the molar ratios of PCL block to PPFA block are calculated to be about 1.49:1 and 2.38:1 for PCL1-*b*-PPFA and PCL2-*b*-PPFA, respectively. The absorption bands at 1790 and 1524 cm^−1^ in the FT-IR spectrum of PCL1-*b*-PPFA (Figure 2b) are assigned to the stretches of reactive ester moiety and pentafluorophenyl group in the PPFA block, respectively. The presence of F 1s signal at the binding energy (BE) of about 690 eV in the XPS wide-scan spectrum of PCL1-*b*-PPFA (Figure 3b) and fluorine signals in the ^19^F NMR spectrum of PCL1-*b*-PPFA (Figure S4a, Supplementary Materials) also indicates that the block copolymers have been successfully synthesized.

The PCL-*b*-PPFA block copolymers were further reacted with 2-aminoethyl-α-d-mannopyranoside via reactive ester-amine reaction, leading to the formation of PCL-*b*-PManEA block copolymers. An excess of 2-aminoethyl-α-d-mannopyranoside was added to ensure complete conversion. The obtained PCL-*b*-PManEA block copolymers from PCL1-*b*-PPFA and PCL2-*b*-PPFA are referred to as PCL1-*b*-PManEA and PCL2-*b*-PManEA, respectively. Figure 1c shows the ^1^H NMR spectrum of PCL1-*b*-PManEA in DMSO-*d*_6_. The chemical shifts in the range of 7.53–7.96 ppm are related to the NH proton of the amide group. The shifts in the region of 2.98–5.18 ppm are attributable to the protons of C-H and OH in carbohydrate and ethylene linker between amide and carbohydrate. Figure 2c shows the FT-IR spectrum of PCL1-*b*-PManEA. The appearance of hydroxyl band above 3000 cm^−1^, the disappearance of reactive ester stretch at 1790 cm^−1^ and the presence of amide peak at 1648 cm^−1^, confirm the successful conversion of reactive ester group into amide group and also the presence of pendant carbohydrate moieties in the PCL-*b*-PManEA block copolymers. XPS analysis of the PCL1-*b*-PManEA copolymers was also carried out to verify the reaction of reactive ester groups of PPFA with 2-aminoethyl-α-d-mannopyranoside. Figure 3c shows the XPS wide-scan spectrum of PCL1-*b*-PManEA. In comparison to the XPS wide-scan spectrum of PCL1-*b*-PPFA precursor (Figure 3b), the appearance of the N 1s signal at the BE of about 400 eV and the disappearance of F 1s signal at the BE of about 690 eV in PCL1-*b*-PManEA (Figure 3c) are consistent with the successful conversion of the reactive ester groups to amide groups.

### 3.2. Preparation of DOX-Loaded PCL-b-PManEA Micelles and Their Lectin Conjugates

The physical entrapment of DOX (one of the common chemotherapeutic agents for intravesical treatment of bladder cancer [28]) into the PCL-*b*-PManEA micelles was performed by a dialysis technique [29]. For efficient encapsulation in the micelles, DOX hydrochloric acid salt (DOX·HCl) is usually deprotonated by triethylamine, which removes the positive charge and enhances hydrophobicity of DOX molecule [30]. The amount of drug encapsulated into the micelles was determined by UV-visible absorption spectroscopy using a calibration curve. A DOX loading of about 3.6 wt % was obtained for the PCL1-*b*-PManEA and PCL2-*b*-PManEA micelles under the same condition.

The size and morphology of DOX-loaded PCL1-*b*-PManEA and PCL2-*b*-PManEA micelles were subsequently analyzed by DLS and FETEM. The hydrodynamic diameter distribution functions of DOX-loaded PCL1-*b*-PManEA and PCL2-*b*-PManEA micelles, obtained from DLS measurements at 90° scattering angle, are shown in Figure S5a,b (Supplementary Materials). DLS results show that DOX-loaded PCL1-*b*-PManEA and PCL2-*b*-PManEA micelles exhibit unimodal size distribution with the mean effective diameter increases from 117.7 to 152.4 nm as the *M*_n_ of PCL block increases from 8000 to 12,800 g/mol. This result indicates that a longer PCL block can lead to a larger micelle core [31]. The observation was further supported by the respective FETEM image of DOX-loaded PCL1-*b*-PManEA and PCL2-*b*-PManEA micelles (Figure S5c,d, Supplementary Materials). From the FETEM image, the DOX-loaded PCL1-*b*-PManEA micelles are close to spherical shape, with an average diameter of about 48 nm. The DOX-loaded PCL2-*b*-PManEA micelles have an average diameter of about 89.2 nm and the micelle morphology is irregular. Furthermore, the average diameter of the micelles observed from FETEM image is smaller than that obtained from DLS measurement. As the FETEM measurements were performed in dry state, the micelles were dehydrated, and the hydrophilic shells collapsed on the hydrophobic cores.

Another objective of present study is to conjugate lectin to the DOX-loaded PCL-*b*-PManEA micelles to enhance their mucoadhesive property. It is known that concanavalin A (ConA) lectin can specifically recognize d-glucopyranoside and d-mannopyranoside moieties [32]. Thus, the d-mannopyranoside moieties in the corona of PCL-*b*-PManEA micelles can readily complex with ConA to produce the DOX-loaded PCL-*b*-PManEA micelles-ConA conjugates (DOX-loaded PCL-*b*-PManEA@ConA micelles). Scheme 2 depicts the conjugation of DOX-loaded PCL-*b*-PManEA micelles with ConA. The successful conjugation was monitored from the evolution of mean effective diameters of DOX-loaded PCL1-*b*-PManEA and PCL2-*b*-PManEA micelles upon binding with ConA (Figure S6, Supplementary Materials). The mean effective diameters of both micelles grow with the increase in conjugation time and reach a plateau after about 5 and 10 min for DOX-loaded PCL1-*b*-PManEA and PCL2-*b*-PManEA micelles, respectively. This phenomenon may be arising from the smaller particle size of PCL1-*b*-PManEA micelles, providing larger surface area for ConA conjugation.

### 3.3. In Vitro Drug Release

**Figure 4:**
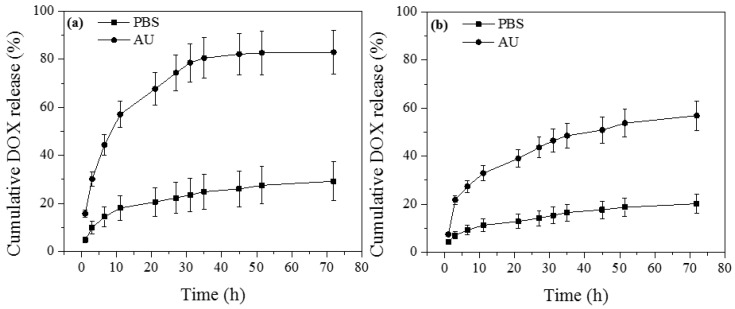
Cumulative DOX release from: (**a**) DOX-loaded PCL1-*b*-PManEA@ConA micelles; and (**b**) DOX-loaded PCL2-*b*-PManEA@ConA micelles in phosphate buffered saline (PBS) and artificial urine (AU).

The drug release from polymeric micelles is frequently studied using a method in which drug-loaded micelles are entrapped inside a dialysis bag and only the released drug could pass through the dialysis membrane [33]. This method was utilized in this study, and the release of DOX from DOX-loaded PCL1-*b*-PManEA and PCL2-*b*-PManEA@ConA micelles was assessed at 37 °C in phosphate buffered saline (PBS, pH = 7.4) and artificial urine (AU, pH = 6.1) for 72 h. The cumulative release curves of DOX are shown in Figure 4. The maximum DOX release in PBS and AU are 29.2% and 82.9% for DOX-loaded PCL1-*b*-PManEA@ConA micelles, and 20.2% and 56.8% for DOX-loaded PCL2-*b*-PManEA@ConA micelles, respectively. In general, the release profiles show an initial burst release of DOX, followed by a sustained and slow release over a prolonged period of time. The initial burst release of DOX from micelles could be attributed to the diffusion of DOX located close to the surface of the micelles, or within the hydrophilic corona, or degradation of the micelles [34]. The total drug released in AU is significantly higher than that in PBS. This phenomenon is due to the higher ionic strength and lower pH of AU [35], which accelerates the hydrolysis of ester moieties in the PCL blocks [36,37]. Furthermore, lower DOX release in both PBS and AU are observed from DOX-loaded PCL2-*b*-PManEA@ConA micelles, which has longer hydrophobic PCL blocks. This result suggests that the release of DOX can be controlled by the hydrophobic PCL chain length, as was also found previously for chitosan-*g*-polydioxane copolymers and oligoagarose-*g*-PCL copolymers [38,39].

### 3.4. Mucoadhesive Study

**Figure 5:**
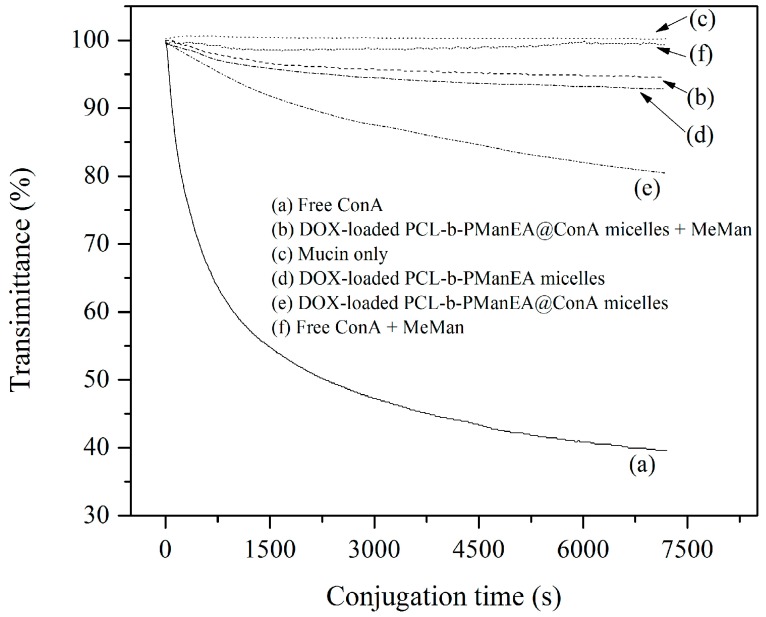
Turbidimetry assay experiment of DOX-loaded PCL1-*b*-PManEA micelles, and free ConA and DOX-loaded PCL1-*b*-PManEA@ConA micelles in the absence and presence of MeMan, with mucin.

The mucoadhesive property of DOX-loaded PCL1-*b*-PManEA@ConA micelles was tested by a turbidimetric assay using mucin from porcine stomach as the model mucin. Upon binding of micelles to mucin, the mucin size will increase, leading to a decrease in the transmittance of the mucin-micelles aqueous solution. Figure 5 shows the turbidimetric assay curves of DOX-loaded PCL1-*b*-PManEA micelles, and free ConA and DOX-loaded PCL1-*b*-PManEA@ConA micelles in the presence and absence of 1-methyl α-d-mannopyranoside (MeMan), with mucin. A control experiment was carried out using mucin or micelles only. Mucin alone does not cause an increase in turbidity (Figure 5, curve c). No obvious increase in turbidity was observed for DOX-loaded PCL1-*b*-PManEA micelles and mucin mixture (Figure 5, curve d). Thus, interaction between the ConA-deficient micelles and mucin is weak or absent. Conversely, the turbidity of DOX-loaded PCL1-*b*-PManEA@ConA micelles and mucin mixture increases within 2 h, suggesting that mucin–ConA interaction has occurred (Figure 5, curve e). After having shown that DOX-loaded PCL1-*b*-PManEA@ConA micelles can interact with mucin, it is important to test the ConA-mediated mucoadhesion via the competitive assays using MeMan, a competitive sugar for ConA [40]. MeMan can compete with the carbohydrate groups in glycoproteins of mucin for binding sites on free ConA and DOX-loaded PCL1-*b*-PManEA@ConA micelles. As shown in Figure 5, the binding of free ConA and DOX-loaded PCL1-*b*-PManEA@ConA micelles to mucin has been effectively inhibited (Figure 5, curve b), indicating the existence of ConA-mediated mucoadhesion. The release profiles together with the ConA-mediated mucoadhesive property of DOX-loaded PCL1-*b*-PManEA@ConA micelles are thus advantageous to sustained intravesical drug delivery.

### 3.5. Cellular Uptake and In Vitro Cytotoxicity Evaluation

**Figure 6:**
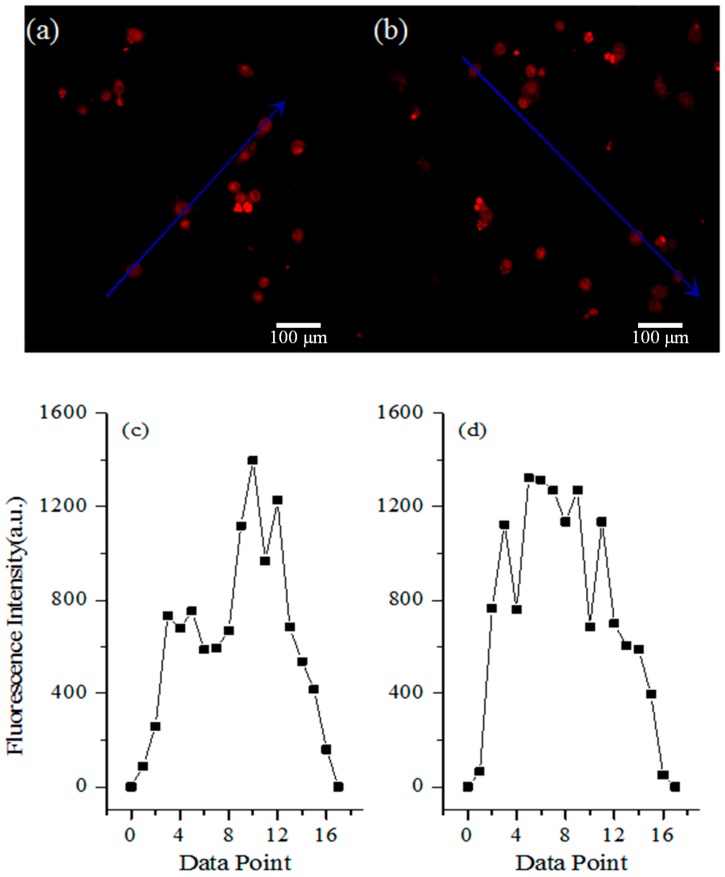
Confocal laser scanning microscope (CLSM) images and fluorescence profiles of UMUC3 cells incubated with: (**a**,**c**) DOX-loaded PCL1-*b*-PManEA@ConA micelles (1 mg/mL); and (**b**,**d**) DOX-loaded PCL1-*b*-PManEA@ConA micelles (1 mg/mL) + MeMan (20 mM) for 2 h.

The in vitro cytotoxicity of DOX-loaded PCL1-*b*-PManEA@ConA micelles against UMUC3 human urothelial carcinoma cell line was compared to that of free DOX and DOX-free PCL1-*b*-PManEA@ConA micelles. The UMUC-3 cells were treated with DOX-loaded PCL1-*b*-PManEA@ConA micelles (1.0 mg/mL) for 2 h. The 2 h cellular incubation time was chosen to imitate the typical intravesical instillation period. The cellular uptake of DOX-loaded PCL1-*b*-PManEA@ConA micelles (1.0 mg/mL) by UMUC-3 cells was first investigated by CLSM. As shown in Figure 6a, the CLSM image shows strong red fluorescence in the cells after just 2 h of incubation with DOX-loaded PCL1-*b*-PManEA@ConA micelles. The red fluorescence is attributed to the internalization of micelles. The fast internalization of micelles may be due to cellular endocytosis or complexation between ConA on micelles and the d-mannose (Man) residues on cell surface glycans [41]. To demonstrate the cellular endocytosis or ConA-mediated cellular internalization of DOX-loaded PCL1-*b*-PManEA@ConA micelles, competitive binding assays were tested using MeMan. MeMan can compete with the Man residues on cell surface glycans for binding sites on DOX-loaded PCL1-*b*-PManEA@ConA micelles. Both MeMan (20 mM) and DOX-loaded PCL1-*b*-PManEA@ConA micelles (1 mg/mL) were cultured together with UMUC-3 cells. In comparison to the CLSM images of the UMUC-3 cells in the absence (Figure 6a) of and presence (Figure 6b) of the MeMan, no obvious decrease in the fluorescence change is observed, suggesting no significant ConA-mediated cellular internalization on the binding of the micelles with UMUC-3 cells. Thus, the interaction of the DOX-loaded PCL1-*b*-PManEA@ConA micelles with UMUC-3 cells is mainly due to the cellular endocytosis.

**Figure 7:**
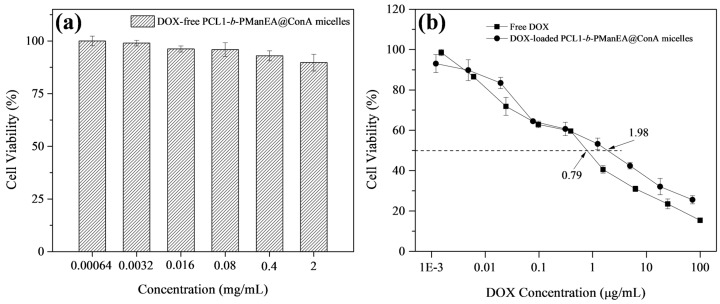
Viability of UMUC3 cells after incubation with: (**a**) DOX-free PCL1-*b*-PManEA@ConA micelles; and (**b**) free DOX and DOX-free PCL1-*b*-PManEA@ConA micelles. Cells were exposed to the free DOX or micelles for 2 h and further cultured with fresh DMEM for 72 h.

The above results have shown that the DOX-loaded PCL1-*b*-PManEA@ConA micelles can be effectively taken up by UMUC-3 cells within 2 h. After additional 72 h of incubation, the in vitro cytotoxicity of free DOX, and DOX-free and DOX-loaded PCL1-*b*-PManEA@ConA micelles are shown in Figure 7. No obvious cytotoxicity of DOX-free PCL1-*b*-PManEA@ConA micelles is observed even at a concentration of 2 mg/mL, indicating that DOX-free PCL1-*b*-PManEA@ConA micelles have excellent biocompatibility and low cytotoxicity towards UMUC-3 cells (Figure 7a). Under the same conditions, DOX-loaded PCL1-*b*-PManEA@ConA micelles show pronounced cytotoxic effects (Figure 7b). Cell viability decreased to 25.6% with DOX-loaded PCL1-*b*-PManEA@ConA micelles concentration of 2 mg/mL. This result is consistent with the cellular uptake results, suggesting that the DOX has been efficiently delivered and released into the nuclei of UMUC-3 cells. The cytotoxicities of free DOX and DOX-loaded PCL1-*b*-PManEA@ConA micelles were further compared. The DOX concentrations that kill 50% of cells (IC_50_) were determined to be 0.79 and 1.98 µg/mL for free DOX and DOX-loaded PCL1-*b*-PManEA@ConA micelles, respectively. Thus, the DOX-loaded PCL1-*b*-PManEA@ConA micelles system with high anti-cancer efficiency, comparable to free DOX, is an advantageous formulation for intravesical treatment of non-muscle invasive (NMI) bladder cancer.

## 4. Conclusions

Amphiphilic PCL-*b*-PManEA block copolymers were synthesized and self-assembled into micelles to encapsulate anticancer drug (DOX) and conjugate ConA lectin. The resulting DOX-loaded PCL-*b*-PManEA@ConA micelles exhibit an initial burst release, followed by a sustained and slow release over three days in an in vitro study. The DOX-loaded PCL-*b*-PManEA@ConA micelles also exhibit mucoadhesive property and enhanced in vitro cytotoxicity against UMUC3 human urothelial carcinoma cells. Thus, the DOX-loaded PCL-*b*-PManEA@ConA micelles have promising applications in intravesical therapy of non-muscle invasive bladder cancer. To further verify the intravesical drug delivery claims in this work, in vivo animal experiments should be performed.

## Supplementary Materials

The following are available online at www.mdpi.com/2073-4360/8/11/379/s1, Table S1: Characteristics of the PCL homopolymers and PCL-*b*-PPFA block copolymers, Figure S1: GPC elution curves of PCL1, PCL2 homopolymers, and PCL1-*b*-PPFA, PCL2-*b*-PPFA block copolymers, Figure S2: ^1^H NMR spectra of (a) PCL2 homopolymer, (b) PCL2-*b*-PPFA and (c) PCL2-*b*-PManEA block copolymers, Figure S3: FT-IR spectra of (a) PCL2 homopolymer, (b) PCL2-*b*-PPFA and (c) PCL2-*b*-PManEA block copolymers, Figure S4: ^19^F NMR spectra of (a) PCL2-*b*-PPFA and (b) PCL2-*b*-PManEA block copolymers, Figure S5: (a,b) Hydrodynamic diameter distribution functions and (c,d) FETEM images of DOX-loaded PCL1-*b*-PManEA and PCL2-*b*-PManEA micelles, respectively, Figure S6: Evolution of mean effective diameters of DOX-loaded PCL1-*b*-PManEA and PCL2-*b*-PManEA micelles upon binding with ConA.
