# Supplementary material for: Conjugation of Lectin to Poly(ε-caprolactone)-block-glycopolymer Micelles for In Vitro Intravesical Drug Delivery
Source: Polymers (Basel). 2016 Oct 26;8(11):379. doi: 10.3390/polym8110379 (PMC6432143; doi:10.3390/polym8110379)
Supplement: Supplementary file 1 [file polymers-08-00379-s001.pdf]

Supplementary Materials: Conjugation of Lectin to Poly(ε-caprolactone)-*block*-glycopolymer Micelles for In Vitro Intravesical Drug Delivery

Ning Ning Li, Xiao Yan Cai, Jiu Cun Chen, Xue Feng Hu and Li Qun Xu

Table S1. Characteristics of the PCL homopolymers and PCL-*b*-PPFA block copolymers

	M_n [a]	PDI [b]	DP [c] of CL		DP of PFA	
			GPC	^{1}H NMR	GPC	^{1}H NMR
PCL1	8,800	1.48	77	97	-	-
PCL2	12,800	1.52	112	140	-	-
PCL1-*b*-PPFA	12,500	1.34	77	97	16	65
PCL2-*b*-PPFA	16,100	1.46	112	140	14	59

[a]: Number-average molecular weight (g/mol) determined by gel permeation chromatography (GPC); [b]: polydispersity index; [c]: Degree of polymerization.

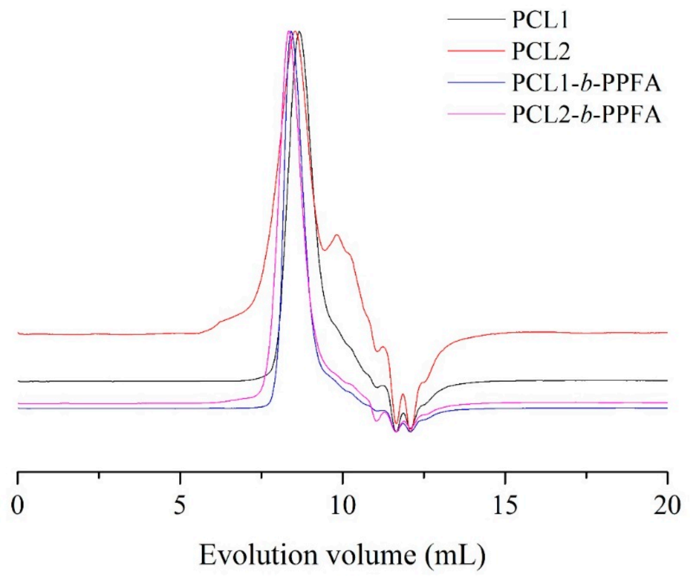

Figure S1. GPC elution curves of PCL1, PCL2 homopolymers, and PCL1-*b*-PPFA, PCL2-*b*-PPFA block copolymers.

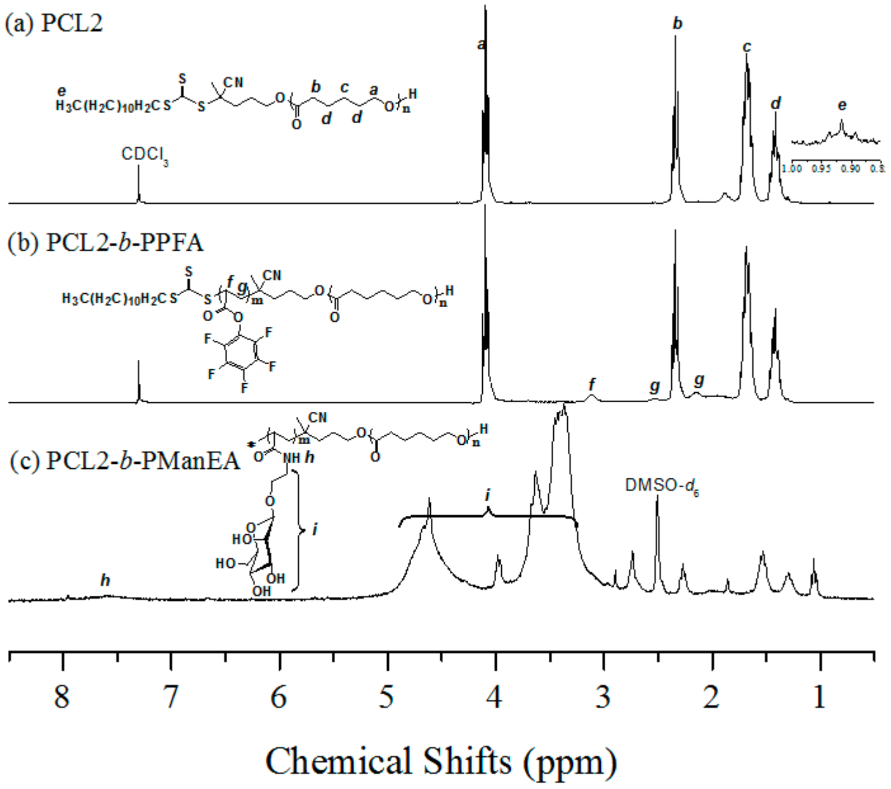

Figure S2. ^{1}H NMR spectra of (**a**) PCL2 homopolymer; (**b**) PCL2-*b*-PPFA and (**c**) PCL2-*b*-PManEA block copolymers.

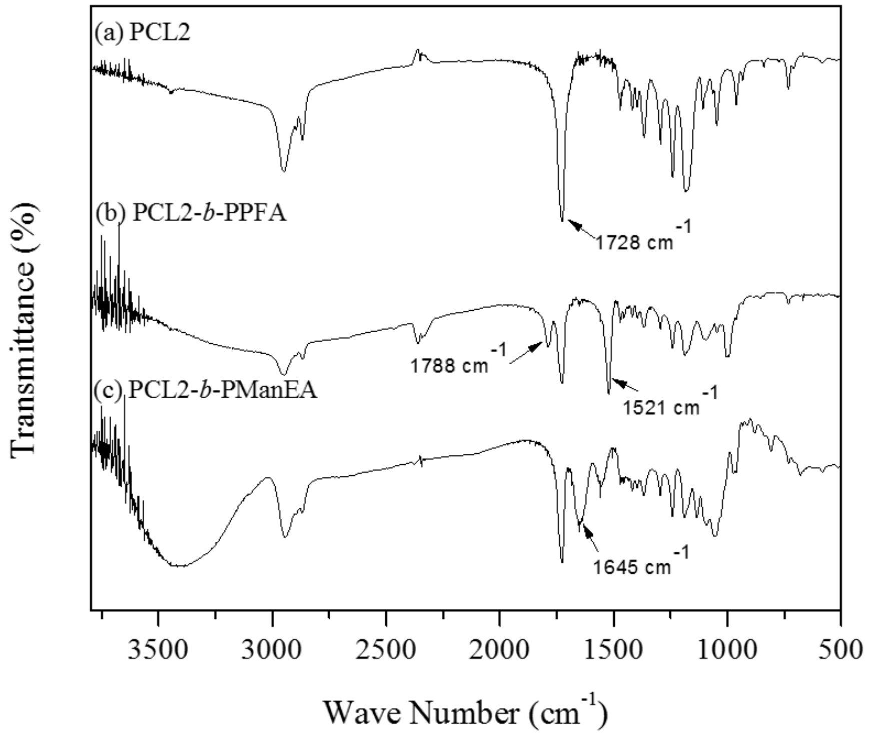

Figure S3. FT-IR spectra of (**a**) PCL2 homopolymer; (**b**) PCL2-*b*-PPFA and (**c**) PCL2-*b*-PManEA block copolymers.

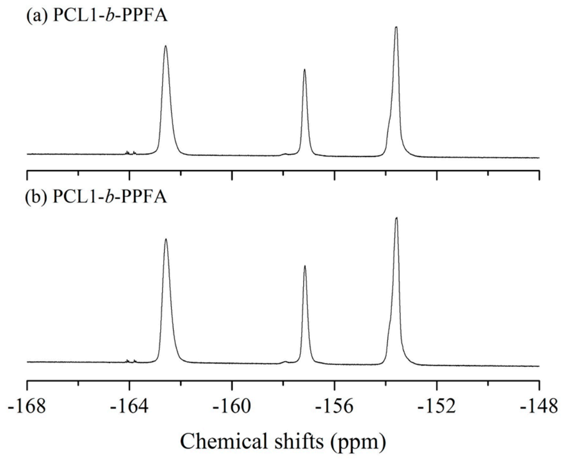

Figure S4. ^{19}F NMR spectra of (**a**) PCL2-*b*-PPFA and (**b**) PCL2-*b*-PMAnEA block copolymers.

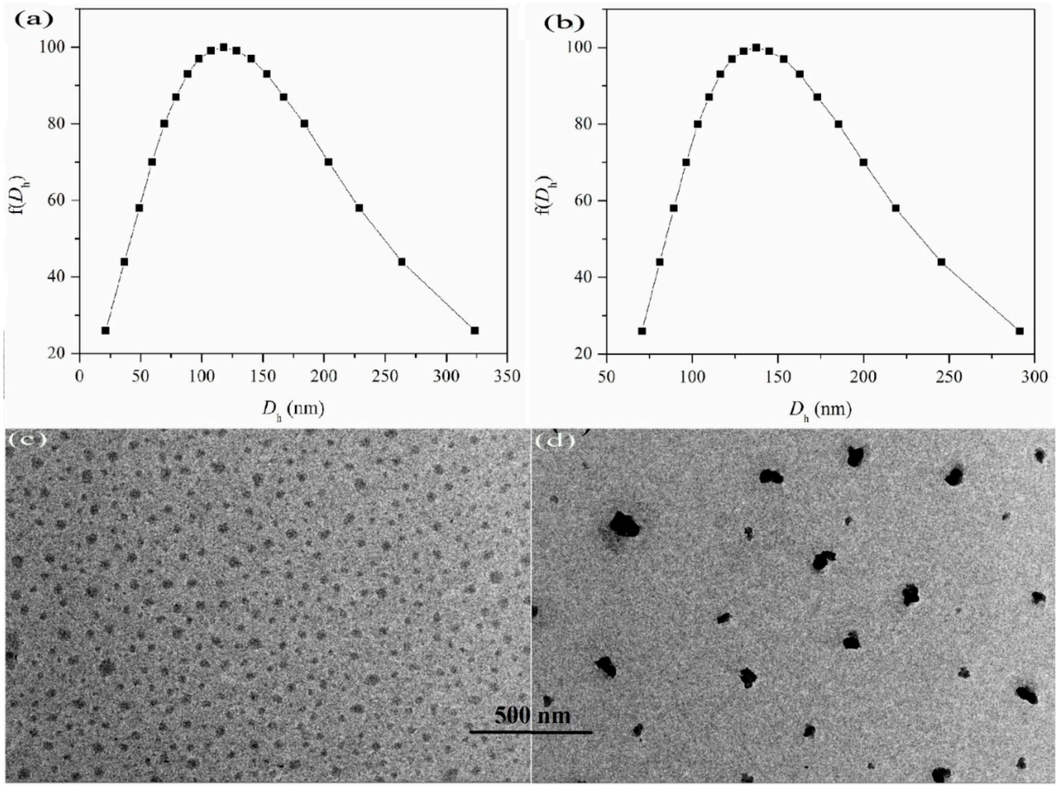

Figure S5. (**a**,**b**) Hydrodynamic diameter distribution functions and (**c**,**d**) FETEM images of DOX-loaded PCL1-*b*-PManEA and PCL2-*b*-PManEA micelles, respectively.

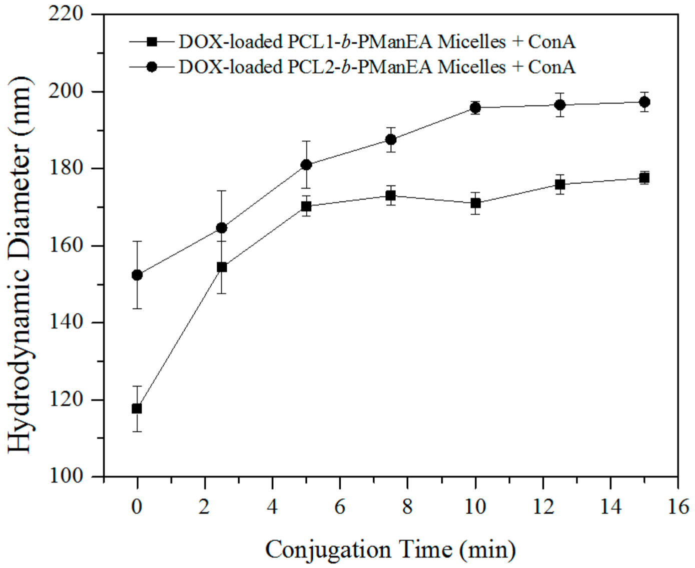

Figure S6. Evolution of mean effective diameters of DOX-loaded PCL1-*b*-PManEA and PCL2-*b*-PManEA micelles upon binding with ConA.
